# The effect of synaptic noise on dendritic morphology

**DOI:** 10.1186/1471-2202-12-S1-P317

**Published:** 2011-07-18

**Authors:** Vandana Reddy Padala, Benjamin Torben-Nielsen, Klaus M Stiefel

**Affiliations:** 1 Theoretical and Experimental Neurobiology Unit, Okinawa Institute of Science and Technology, Okinawa, Japan; 2 Edmond and Lily Safra Center for Brain Sciences, Hebrew University, Jerusalem; 3 Department of Neurobiology, Hebrew University, Jerusalem

## 

Neurons exhibit enormous diversity in their dendritic morphology and the highly structured dendrites help neurons to perform various computations. The complex relationship between the dendrite morphology and their emergent computational functionality is not well understood and is an active area of research. To study this relationship we previously developed an inverse approach [1] in which model neurons, including dendritic morphology, are optimized to perform a certain computational function. The synaptic inputs in the earlier study were deterministic. However, synaptic noise is known to have profound effects on computations performed by neurons [2].

In this work we investigate the influence of synaptic noise on the optimal morphology to perform a computational task. We consider two types of synaptic noise, namely spatial and temporal noise. The former relates to failure in synaptic transmission while the latter relates to temporal jitter of events. We studied the effect of both types of noise in the input-order detection task. In this task, synaptic inputs are inserted into a model neuron when the dendrites of the model “grow” into two spatially separate regions; the number of synapses depends on the dendritic length in that region. Synapses in both groups are activated sequentially with some interval (Δt) between them. The model neuron is then optimized to respond strongly to a particular order of synaptic activation (for instance, region 1 before region 2) and not in the reverse order. We implement spatial synaptic noise as a probability of synaptic transmission failure, i.e., not all synapses will actually transmit a signal. Temporal synaptic noise is implemented as temporal jitter in the activation of particular synapses. We applied both types of noise in separation and analyzed the resultant morphological structures. Thus, we sought for a morphological mechanism that allows neurons to cope with synaptic noise.

We found that different types of synaptic noise yielded different influences on the dendritic morphologies and to different extents. We also compared the results to Nucleus Laminaris neurons--which are assumed to perform a task similar to input-order detection-- and found that model neurons optimized to cope with synaptic noise showed stronger similarity to these neurons than model neurons optimized with deterministic inputs.
